# Bis{tris­[3-(2-pyrid­yl)-1*H*-pyrazole]nickel(II)} dodeca­molybdo(V,VI)phosphate hexa­hydrate

**DOI:** 10.1107/S1600536810005945

**Published:** 2010-02-20

**Authors:** Lujiang Hao, Tongjun Liu, Jiangkui Chen, Xiaofei Zhang

**Affiliations:** a College of Food and Biological Engineering, Shandong Institute of Light Industry, Jinan 250353, People’s Republic of China

## Abstract

The hydro­thermally prepared title compound, [Ni(C_8_H_7_N_3_)_3_]_2_[PMo_12_O_40_]·6H_2_O, is a member of the isotypic series [(*M*(C_8_H_7_N_3_)_3_]_2_[PMo_12_O_40_]·6H_2_O where *M* is Mn, Cd, and Fe. The Ni^2+^ cation is in a distorted octa­hedral environment, coordinated by six N atoms from three chelating 3-(2-pyrid­yl)-1*H*-pyrazole ligands. In the one-electron reduced heteropolyanion, two O atoms of the central PO_4_ group (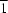 symmetry) are equally disordered about an inversion centre. N—H⋯O and O—H⋯O hydrogen bonds contribute to the crystal packing. Compared with the isotypic structures, the main difference is related with the *M*—N bond lengths, whereas all other bond lengths, angles and the hydrogen-bonding motifs are very similar.

## Related literature

For the isotypic analogues, see: Hao, Ma *et al.* (2010 ▶) for *M* = Mn; Hao, Wang *et al.* (2010 ▶) for *M* = Cd; Hao, Liu, *et al.* (2010 ▶) for *M* = Fe. For general background to polyoxometalates, see: Pope & Müller (1991 ▶). For polyoxometalates modified with amines, see: Zhang, Dou *et al.* (2009 ▶); Zhang, Wei *et al.* (2009 ▶). For the structures of other reduced heteropolyanions with composition [PMo_12_O_40_]^4−^, see: Artero & Proust (2000 ▶); Kurmoo *et al.* (1998 ▶).; Niu *et al.* (1999 ▶). For the role of amines in hydro­thermal synthesis, see: Yang *et al.* (2003 ▶). 
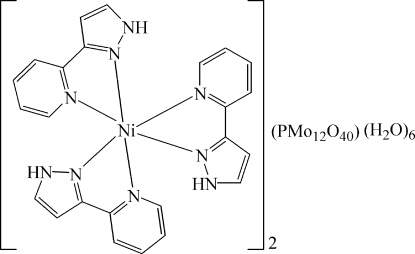


## Experimental

### 

#### Crystal data

[Ni(C_8_H_7_N_3_)_3_]_2_[PMo_12_O_40_]·6H_2_O
*M* *_r_* = 2918.76
Monoclinic, 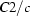

*a* = 18.741 (4) Å
*b* = 16.285 (3) Å
*c* = 27.678 (6) Å
β = 103.83 (3)°
*V* = 8202 (3) Å^3^
*Z* = 4
Mo *K*α radiation
μ = 2.34 mm^−1^
*T* = 293 K
0.42 × 0.27 × 0.20 mm

#### Data collection

Bruker APEXII CCD diffractometer
Absorption correction: multi-scan (*SADABS*; Bruker, 2001 ▶) *T*_min_ = 0.440, *T*_max_ = 0.652
22802 measured reflections
7216 independent reflections
5310 reflections with *I* > 2σ(*I*)
*R*_int_ = 0.062

#### Refinement

*R*[*F* ^2^ > 2σ(*F* ^2^)] = 0.053
*wR*(*F* ^2^) = 0.155
*S* = 1.00
7216 reflections
592 parameters
18 restraints
H-atom parameters constrained
Δρ_max_ = 1.58 e Å^−3^
Δρ_min_ = −0.67 e Å^−3^

### 

Data collection: *APEX2* (Bruker, 2004 ▶); cell refinement: *SAINT-Plus* (Bruker, 2001 ▶); data reduction: *SAINT-Plus*; program(s) used to solve structure: *SHELXS97* (Sheldrick, 2008 ▶); program(s) used to refine structure: *SHELXL97* (Sheldrick, 2008 ▶); molecular graphics: *SHELXTL* (Sheldrick, 2008 ▶); software used to prepare material for publication: *SHELXTL*.

## Supplementary Material

Crystal structure: contains datablocks global, I. DOI: 10.1107/S1600536810005945/wm2306sup1.cif

Structure factors: contains datablocks I. DOI: 10.1107/S1600536810005945/wm2306Isup2.hkl

Additional supplementary materials:  crystallographic information; 3D view; checkCIF report

## Figures and Tables

**Table 1:** Selected bond lengths (Å)

Ni1—N5	2.077 (19)
Ni1—N8	2.06 (2)
Ni1—N2	2.084 (19)
Ni1—N4	2.13 (2)
Ni1—N1	2.118 (17)
Ni1—N7	2.14 (2)
P1—O21*A*^i^	1.49 (2)
P1—O21*B*^i^	1.50 (3)
P1—O19*B*^i^	1.55 (3)
P1—O19*A*^i^	1.57 (3)

Symmetry code: (i) 
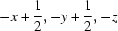
.

**Table 2:** Hydrogen-bond geometry (Å, °)

*D*—H⋯*A*	*D*—H	H⋯*A*	*D*⋯*A*	*D*—H⋯*A*
N3—H3*A*⋯O17^ii^	0.86	2.05	2.83 (3)	149
N6—H6⋯O2*W*	0.86	1.99	2.84 (5)	166
N9—H9*A*⋯O1*W*	0.86	1.92	2.74 (3)	160

Symmetry code: (ii) 
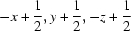
.

**Fig. 1.**
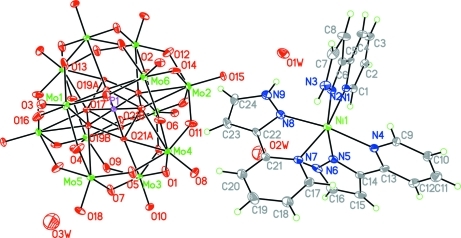
The building blocks of the title compound with the atom-labelling scheme. Displacement ellipsoids are drawn at the 30% probability level; H atoms are given as spheres of arbitrary radius.

**Fig. 2.**
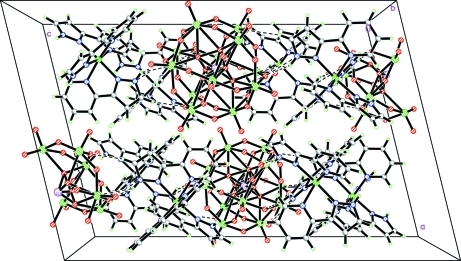
The crystal packing of the title compound, displayed with N—H···O and O—H···O hydrogen bonds as dashed lines.

## supplementary crystallographic information

### Comment

The design and synthesis of polyoxometalates has attracted continuous research interest not only because of their appealing structural and topological novelties, but also due to their interesting optical, electronic, magnetic, and catalytic properties, as well as their potential medical applications (Pope & Müller, 1991). In our research group, organic amines, such as 3-(2-pyridyl)pyrazole and pyrazine, are used to effectively modify polyoxomolybdates under hydrothermal condictions (Zhang, Dou *et al.*, 2009; Zhang, Wei *et al.*, 2009). Here, we describe the synthesis and structural characterization of the title compound.

As shown in Figure 1, the asymmetric unit of the title compound consists of three subunits, *viz.* of a complex [Ni(C_8_H_7_N_3_)_3_]^2+^ cation, half of a heteropolyanion [PMo_12_O_40_]^4-^ and of three uncoordinated water molecules. The nickel(II) ion is in a distorted octahedral coordination by six N atoms from three chelating 3-(2-pyridyl)-1*H*-pyrazole ligands.

The heteropolyanion [PMo_12_O_40_]^4-^ anion is a one-electron reduced derivative of [PMo_12_O_40_]^3-^, similar to anions with different counter cations as reported by Artero & Proust (2000); Kurmoo *et al.* (1998); Niu *et al.* (1999). The employed organic ligand appears to adjust the pH value, and additionally supplies reducing electrons, which is a commonly observed feature of hydrothermal syntheses when organic amines are used to prepare various hybrid materials, zeolites or metal phosphates (Yang *et al.*, 2003).

In the Keggin-type heteropolyanion, each Mo atom is surrounded by six O atoms and the P atom is located at the center of the anion. There are four kinds of O atoms present in the anion according to their coordination environments: O*_a_* (O atoms in the disordered PO_4_ tetrahedron), O*_b_* (bridging O atoms between two triplet groups of MoO_6_ octahedra), O*_c_* (bridging O atoms within one triplet group of MoO_6_ octahedra) and O*_d_* (terminal O atoms). The P—O bond distances are in the normal range of 1.49 (2)—1.57 (3) Å. The Mo—O bond distances vary widely from 1.653 (15) to 2.55 (2) Å. The shortest Mo—O bonds are in the range of 1.653 (15)—1.665 (16) Å for the terminal oxygen atoms. The longest Mo—O lengths are in the range of 2.44 (2)—2.55 (2) Å for those oxygen atoms connected with both Mo and P atoms.

N—H···O and O—H···O hydrogen bonding between the cationic and anionic moieties and the uncoordinated water molecules leads to a consolidation of the structure (Fig. 2; Table 2).

The crystal structure of [(Ni(C_8_H_7_N_3_)_3_]_2_[PMo_12_O_40_](H_2_O)_6_ is isotypic with the Mn^2+^, Cd^2+^, and Fe^2+^ analogues, [(Mn(C_8_H_7_N_3_)_3_]_2_[PMo_12_O_40_](H_2_O)_6_ (Hao, Ma *et al.* (2010).), [(Cd(C_8_H_7_N_3_)_3_]_2_[PMo_12_O_40_](H_2_O)_6_ (Hao, Wang *et al.* (2010).), [(Fe(C_8_H_7_N_3_)_3_]_2_[PMo_12_O_40_](H_2_O)_6_ (Hao, Liu *et al.*, 2010). In comparison with the Mn^2+^, Cd^2+^, and Fe^2+^ analogues, the Ni—N bond lengths are somewhat shorter at 2.077 (19)—2.14 (2) Å, versus 2.224 (6)—2.283 (5) Å for Mn—N, 2.085 (19)—2.15 (2) Å for Fe—N, and 2.316 (7)—2.334 (6) Å for Cd—N, whereas all other bond lengths and angles and the hydrogen-bonding motifs are very similar in the four structures.

### Experimental

A mixture of 3-(2-pyridyl)-1*H*-pyrazole (0.5 mmoL 0.07 g), sodium molybdate (0.4 mmoL, 0.10 g), nickel(II) chloride hexahydrate (0.25 mmol, 0.05 g), and dipotassium hydrogenphosphate (0.22 mmol, 0.05 g) in 10 ml distilled water was sealed in a 25 ml Teflon-lined stainless steel autoclave and was kept at 433 K for three days. Green crystals suitable for the X-ray experiment were obtained. IR(cm^-1^): 3376, 3136, 2961, 1614, 1568, 1522, 1457, 1439, 1364, 1300, 1097, 950, 913, 812, 636, 507.

TGA curve shows a separation of lattice water molecules and the organic ligands above 343 and 682 K, respectively. The overall thermal decomposition process can be described by the followed equation: 4C_48_H_54_Ni_2_Mo_12_N_18_O_46_P + 325O_2_ = 108H_2_O + 192CO_2_ + 36N_2_O_5_ + 8NiO + 2P_2_O_5_ + 48MoO_3_

### Refinement

All hydrogen atoms bound to aromatic carbon atoms were refined in calculated positions using a riding model with a C—H distance of 0.93 Å and *U*_iso_ = 1.2*U*_eq_(C). Hydrogen atoms attached to aromatic N atoms were refined with a N—H distance of 0.86 Å and *U*_iso_ = 1.2*U*_eq_(N). The hydrogen atoms of the three uncoordinated water molecules could not be located unambiguously from difference Fourier maps, probably due to disorder of the water molecules. Thus the structure was refined without the H atoms of the water molecules (which includes the water O atoms O1W, O2W, O3W). In the PO_4_ unit, the two oxygen atoms (O19 and O21) are equally disordered about the inversion centre. In the final difference Fourier map the highest peak is 2.70 Å from atom O2w and the deepest hole is 1.25 Å from atom O12. The highest peak is located in the voids of the crystal structure and may be associated with an additional water molecule. However, refinement of this position did not result in a reasonable model. Hence this position was also excluded from the final refinement.

### Crystal data

**Table:**

[Ni(C_8_H_7_N_3_)_3_]_2_[PMo_12_O_40_]·6H_2_O	*F*(000) = 5644
*M**_r_* = 2918.76	*D*_x_ = 2.364 Mg m^−^^3^
Monoclinic, *C*2/*c*	Mo *K*α radiation, λ = 0.71073 Å
Hall symbol: -C 2yc	Cell parameters from 7216 reflections
*a* = 18.741 (4) Å	θ = 1.7–25.0°
*b* = 16.285 (3) Å	µ = 2.34 mm^−^^1^
*c* = 27.678 (6) Å	*T* = 293 K
β = 103.83 (3)°	Block, green
*V* = 8202 (3) Å^3^	0.42 × 0.27 × 0.20 mm
*Z* = 4	

### Data collection

**Table:**

Bruker APEXII CCD diffractometer	7216 independent reflections
Radiation source: fine-focus sealed tube	5310 reflections with *I* > 2σ(*I*)
graphite	*R*_int_ = 0.062
phi and ω scans	θ_max_ = 25.0°, θ_min_ = 1.7°
Absorption correction: multi-scan (*SADABS*; Bruker, 2001)	*h* = −22→22
*T*_min_ = 0.440, *T*_max_ = 0.652	*k* = −17→19
22802 measured reflections	*l* = −32→32

### Refinement

**Table:**

Refinement on *F*^2^	Primary atom site location: structure-invariant direct methods
Least-squares matrix: full	Secondary atom site location: difference Fourier map
*R*[*F*^2^ > 2σ(*F*^2^)] = 0.053	Hydrogen site location: inferred from neighbouring sites
*wR*(*F*^2^) = 0.155	H-atom parameters constrained
*S* = 1.00	*w* = 1/[σ^2^(*F*_o_^2^) + (0.088*P*)^2^ + 10.3967*P*] where *P* = (*F*_o_^2^ + 2*F*_c_^2^)/3
7216 reflections	(Δ/σ)_max_ = 0.001
592 parameters	Δρ_max_ = 1.58 e Å^−^^3^
18 restraints	Δρ_min_ = −0.67 e Å^−^^3^

### Special details

**Table:**

Geometry. All esds (except the esd in the dihedral angle between two l.s. planes) are estimated using the full covariance matrix. The cell esds are taken into account individually in the estimation of esds in distances, angles and torsion angles; correlations between esds in cell parameters are only used when they are defined by crystal symmetry. An approximate (isotropic) treatment of cell esds is used for estimating esds involving l.s. planes.
Refinement. Refinement of *F*^2^ against ALL reflections. The weighted *R*-factor wR and goodness of fit *S* are based on *F*^2^, conventional *R*-factors *R* are based on *F*, with *F* set to zero for negative *F*^2^. The threshold expression of *F*^2^ > σ(*F*^2^) is used only for calculating *R*-factors(gt) etc. and is not relevant to the choice of reflections for refinement. *R*-factors based on *F*^2^ are statistically about twice as large as those based on *F*, and *R*- factors based on ALL data will be even larger.

### Fractional atomic coordinates and isotropic or equivalent isotropic displacement parameters (Å^2^)

**Table:**

	*x*	*y*	*z*	*U*_iso_*/*U*_eq_	Occ. (<1)
C1	0.2023 (14)	0.8990 (15)	0.0923 (9)	0.049 (6)	
H1	0.2445	0.8942	0.0803	0.059*	
C2	0.1420 (14)	0.9332 (15)	0.0634 (8)	0.051 (6)	
H2	0.1428	0.9526	0.0320	0.062*	
C3	0.0798 (15)	0.9396 (17)	0.0799 (9)	0.058 (7)	
H3	0.0379	0.9631	0.0598	0.070*	
C4	0.0790 (14)	0.9114 (18)	0.1261 (10)	0.061 (7)	
H4	0.0365	0.9150	0.1378	0.073*	
C5	0.1416 (12)	0.8778 (14)	0.1549 (8)	0.040 (5)	
C6	0.1465 (13)	0.8481 (14)	0.2052 (8)	0.043 (6)	
C7	0.0946 (16)	0.845 (2)	0.2344 (11)	0.074 (9)	
H7	0.0455	0.8612	0.2257	0.089*	
C8	0.135 (2)	0.813 (2)	0.2804 (11)	0.074 (9)	
H8	0.1172	0.8038	0.3086	0.089*	
C9	0.3232 (13)	0.9754 (14)	0.2567 (9)	0.047 (6)	
H9	0.2895	0.9552	0.2735	0.056*	
C10	0.3547 (16)	1.050 (2)	0.2699 (11)	0.061 (8)	
H10	0.3434	1.0793	0.2959	0.073*	
C11	0.4016 (19)	1.080 (2)	0.2455 (15)	0.090 (11)	
H11	0.4226	1.1315	0.2542	0.108*	
C12	0.4194 (14)	1.0371 (19)	0.2079 (10)	0.063 (7)	
H12	0.4523	1.0577	0.1906	0.075*	
C13	0.3872 (13)	0.9634 (16)	0.1968 (9)	0.052 (6)	
C14	0.4049 (13)	0.9098 (17)	0.1581 (9)	0.051 (6)	
C15	0.4550 (15)	0.919 (2)	0.1271 (12)	0.075 (10)	
H15	0.4876	0.9621	0.1266	0.090*	
C16	0.4433 (17)	0.848 (2)	0.0973 (12)	0.074 (9)	
H16	0.4670	0.8339	0.0726	0.089*	
C17	0.4107 (15)	0.7609 (19)	0.2864 (10)	0.060 (7)	
H17	0.4164	0.8166	0.2938	0.072*	
C18	0.4501 (18)	0.706 (2)	0.3200 (13)	0.082 (10)	
H18	0.4809	0.7241	0.3496	0.099*	
C19	0.443 (2)	0.625 (2)	0.3091 (14)	0.092 (11)	
H19	0.4703	0.5868	0.3308	0.110*	
C20	0.3948 (17)	0.5994 (19)	0.2644 (12)	0.078 (9)	
H20	0.3882	0.5440	0.2564	0.094*	
C21	0.3580 (14)	0.6586 (16)	0.2337 (10)	0.047 (6)	
C22	0.3041 (14)	0.6382 (16)	0.1873 (9)	0.048 (6)	
C23	0.2838 (17)	0.5645 (17)	0.1627 (10)	0.063 (8)	
H23	0.3033	0.5127	0.1717	0.075*	
C24	0.2287 (18)	0.5843 (17)	0.1221 (11)	0.069 (8)	
H24	0.2030	0.5479	0.0984	0.083*	
Ni1	0.29253 (16)	0.81664 (19)	0.19077 (11)	0.0411 (8)	
Mo1	0.24269 (12)	0.14134 (13)	0.11084 (7)	0.0420 (6)	
Mo2	0.19263 (12)	0.45800 (12)	−0.01469 (8)	0.0417 (6)	
Mo3	0.42259 (10)	0.31668 (12)	−0.01927 (7)	0.0370 (6)	
Mo4	0.35304 (11)	0.40513 (13)	0.07783 (7)	0.0388 (6)	
Mo5	0.41654 (10)	0.19716 (13)	0.08814 (7)	0.0402 (6)	
Mo6	0.17514 (11)	0.34583 (13)	0.09260 (7)	0.0385 (6)	
N1	0.2031 (10)	0.8709 (11)	0.1392 (6)	0.038 (4)	
N2	0.2102 (10)	0.8191 (12)	0.2297 (7)	0.043 (5)	
N3	0.2020 (13)	0.7966 (15)	0.2756 (8)	0.056 (6)	
H3A	0.2361	0.7749	0.2984	0.067*	
N4	0.3392 (11)	0.9302 (13)	0.2204 (8)	0.049 (5)	
N5	0.3682 (11)	0.8410 (13)	0.1487 (7)	0.048 (5)	
N6	0.3924 (12)	0.8050 (16)	0.1113 (8)	0.066 (7)	
H6	0.3760	0.7588	0.0981	0.079*	
N7	0.3642 (10)	0.7388 (13)	0.2433 (7)	0.048 (5)	
N8	0.2643 (11)	0.7011 (13)	0.1619 (7)	0.050 (5)	
N9	0.2184 (12)	0.6674 (15)	0.1229 (8)	0.062 (6)	
H9A	0.1865	0.6941	0.1011	0.074*	
O1	0.4066 (11)	0.3947 (10)	0.0246 (6)	0.063 (5)	
O2	0.1446 (9)	0.3914 (12)	0.1373 (6)	0.057 (5)	
O3	0.2405 (9)	0.0897 (11)	0.1619 (6)	0.055 (5)	
O4	0.3441 (11)	0.1729 (13)	0.1236 (8)	0.079 (7)	
O5	0.4024 (11)	0.3112 (10)	0.1052 (7)	0.065 (6)	
O6	0.2780 (9)	0.3874 (13)	0.1090 (7)	0.066 (5)	
O7	0.4526 (11)	0.2417 (10)	0.0345 (6)	0.060 (5)	
O8	0.4028 (11)	0.4785 (11)	0.1120 (7)	0.066 (5)	
O9	0.4027 (11)	0.2166 (11)	−0.0602 (7)	0.082 (7)	
O10	0.5030 (9)	0.3438 (11)	−0.0293 (7)	0.061 (5)	
O11	0.2863 (9)	0.4671 (14)	0.0283 (7)	0.080 (7)	
O12	0.1059 (11)	0.4001 (13)	−0.0525 (7)	0.081 (7)	
O13	0.1431 (12)	0.1324 (11)	0.0721 (8)	0.086 (8)	
O14	0.1575 (9)	0.4236 (14)	0.0422 (7)	0.071 (6)	
O15	0.1673 (10)	0.5557 (10)	−0.0206 (7)	0.062 (5)	
O16	0.2676 (12)	0.0575 (14)	0.0694 (8)	0.090 (7)	
O17	0.2203 (11)	0.2506 (11)	0.1275 (7)	0.074 (6)	
O18	0.4935 (9)	0.1738 (12)	0.1293 (6)	0.064 (5)	
O19A	0.2028 (14)	0.2368 (16)	0.0390 (10)	0.029 (6)	0.50
O21A	0.3246 (13)	0.2799 (16)	0.0253 (10)	0.028 (6)	0.50
O19B	0.2904 (15)	0.1847 (16)	0.0376 (9)	0.031 (6)	0.50
O21B	0.2482 (14)	0.3299 (17)	0.0264 (9)	0.032 (6)	0.50
O1W	0.1158 (13)	0.7172 (16)	0.0399 (9)	0.099 (8)	
O2W	0.358 (2)	0.653 (2)	0.0612 (15)	0.163 (12)	
O3W	0.534 (3)	0.072 (3)	0.027 (2)	0.27 (2)	
P1	0.2500	0.2500	0.0000	0.0252 (15)	

### Atomic displacement parameters (Å^2^)

**Table:**

	*U*^11^	*U*^22^	*U*^33^	*U*^12^	*U*^13^	*U*^23^
C1	0.054 (15)	0.049 (15)	0.045 (15)	0.001 (12)	0.013 (12)	0.011 (12)
C2	0.058 (17)	0.055 (15)	0.035 (12)	−0.005 (13)	0.000 (12)	0.017 (11)
C3	0.045 (16)	0.075 (19)	0.047 (15)	−0.005 (14)	−0.005 (12)	0.010 (14)
C4	0.037 (15)	0.09 (2)	0.055 (16)	0.006 (14)	0.003 (12)	0.004 (15)
C5	0.032 (12)	0.038 (13)	0.048 (14)	−0.011 (10)	0.007 (10)	0.003 (11)
C6	0.042 (14)	0.049 (14)	0.041 (13)	−0.002 (11)	0.013 (11)	0.006 (11)
C7	0.047 (17)	0.11 (3)	0.07 (2)	0.013 (17)	0.020 (15)	0.014 (19)
C8	0.09 (2)	0.09 (2)	0.053 (19)	−0.016 (19)	0.028 (18)	0.012 (16)
C9	0.052 (15)	0.043 (14)	0.045 (14)	−0.003 (11)	0.009 (11)	0.000 (11)
C10	0.065 (19)	0.06 (2)	0.053 (17)	−0.003 (16)	0.003 (14)	−0.001 (16)
C11	0.08 (2)	0.09 (2)	0.09 (3)	−0.001 (19)	−0.01 (2)	0.02 (2)
C12	0.055 (16)	0.08 (2)	0.051 (16)	−0.007 (15)	0.005 (13)	0.019 (15)
C13	0.034 (13)	0.062 (17)	0.054 (15)	−0.010 (12)	−0.003 (11)	0.016 (13)
C14	0.038 (13)	0.061 (17)	0.053 (15)	0.002 (12)	0.008 (11)	0.016 (13)
C15	0.040 (16)	0.10 (3)	0.08 (2)	0.005 (16)	0.010 (15)	0.05 (2)
C16	0.06 (2)	0.10 (2)	0.07 (2)	0.017 (17)	0.039 (16)	0.033 (18)
C17	0.057 (17)	0.06 (2)	0.057 (17)	−0.006 (15)	0.006 (14)	−0.005 (15)
C18	0.08 (2)	0.08 (2)	0.07 (2)	0.009 (19)	−0.025 (18)	0.012 (19)
C19	0.10 (3)	0.08 (3)	0.09 (3)	0.03 (2)	0.00 (2)	0.03 (2)
C20	0.07 (2)	0.07 (2)	0.08 (2)	0.008 (16)	−0.015 (17)	0.004 (17)
C21	0.046 (15)	0.054 (16)	0.042 (17)	0.008 (12)	0.010 (12)	0.005 (13)
C22	0.049 (15)	0.049 (16)	0.046 (14)	0.017 (13)	0.016 (12)	0.000 (13)
C23	0.08 (2)	0.052 (17)	0.052 (16)	0.023 (15)	0.014 (15)	−0.005 (13)
C24	0.09 (2)	0.053 (17)	0.061 (19)	−0.007 (16)	0.023 (17)	−0.034 (14)
Ni1	0.0353 (16)	0.0469 (18)	0.0408 (17)	0.0041 (13)	0.0083 (13)	0.0042 (14)
Mo1	0.0552 (13)	0.0425 (12)	0.0281 (10)	−0.0059 (10)	0.0094 (9)	0.0041 (9)
Mo2	0.0455 (12)	0.0313 (11)	0.0521 (13)	0.0059 (9)	0.0190 (10)	0.0026 (9)
Mo3	0.0258 (10)	0.0433 (12)	0.0418 (12)	−0.0038 (8)	0.0081 (8)	−0.0004 (9)
Mo4	0.0324 (11)	0.0423 (12)	0.0400 (11)	−0.0070 (9)	0.0055 (8)	−0.0093 (9)
Mo5	0.0286 (11)	0.0528 (13)	0.0353 (11)	0.0018 (9)	0.0003 (8)	0.0032 (9)
Mo6	0.0400 (12)	0.0457 (12)	0.0304 (10)	0.0085 (9)	0.0095 (8)	−0.0063 (9)
N1	0.035 (10)	0.043 (11)	0.037 (10)	0.002 (8)	0.009 (8)	0.009 (8)
N2	0.040 (11)	0.054 (12)	0.034 (10)	0.004 (9)	0.008 (9)	0.005 (9)
N3	0.049 (14)	0.074 (17)	0.043 (13)	−0.003 (12)	0.010 (11)	0.015 (12)
N4	0.041 (12)	0.047 (13)	0.058 (13)	0.000 (10)	0.009 (10)	0.014 (11)
N5	0.041 (12)	0.061 (14)	0.048 (12)	0.009 (10)	0.021 (10)	0.015 (10)
N6	0.053 (14)	0.084 (18)	0.061 (14)	0.029 (13)	0.014 (12)	0.010 (13)
N7	0.037 (11)	0.058 (14)	0.047 (12)	0.004 (10)	0.008 (9)	0.006 (10)
N8	0.054 (13)	0.056 (13)	0.036 (11)	0.008 (10)	0.003 (10)	−0.006 (10)
N9	0.059 (15)	0.075 (18)	0.047 (13)	0.006 (12)	0.004 (11)	0.005 (12)
O1	0.100 (15)	0.048 (11)	0.055 (11)	0.024 (10)	0.045 (10)	0.010 (9)
O2	0.050 (10)	0.078 (14)	0.045 (10)	0.003 (9)	0.018 (8)	−0.018 (9)
O3	0.058 (11)	0.069 (12)	0.035 (9)	−0.003 (9)	0.005 (8)	0.018 (8)
O4	0.073 (14)	0.094 (16)	0.087 (15)	−0.045 (12)	0.052 (12)	−0.053 (12)
O5	0.104 (16)	0.049 (11)	0.055 (11)	0.026 (10)	0.044 (11)	0.012 (9)
O6	0.028 (9)	0.102 (15)	0.067 (12)	0.007 (9)	0.008 (8)	0.041 (11)
O7	0.095 (14)	0.045 (10)	0.050 (10)	0.022 (10)	0.041 (10)	0.008 (8)
O8	0.077 (13)	0.056 (12)	0.060 (13)	−0.024 (10)	0.009 (10)	−0.021 (10)
O9	0.091 (15)	0.043 (11)	0.077 (13)	0.018 (10)	−0.048 (11)	−0.013 (10)
O10	0.043 (10)	0.061 (12)	0.091 (14)	−0.010 (9)	0.038 (10)	−0.002 (10)
O11	0.038 (10)	0.129 (18)	0.077 (14)	0.007 (11)	0.021 (9)	0.057 (13)
O12	0.079 (14)	0.096 (16)	0.087 (14)	−0.047 (12)	0.058 (12)	−0.060 (12)
O13	0.095 (15)	0.036 (10)	0.089 (15)	0.017 (10)	−0.051 (12)	−0.014 (10)
O14	0.027 (9)	0.118 (17)	0.067 (13)	0.004 (10)	0.012 (9)	0.050 (12)
O15	0.064 (12)	0.031 (9)	0.090 (15)	0.011 (8)	0.015 (11)	0.012 (10)
O16	0.082 (13)	0.102 (15)	0.102 (14)	−0.047 (12)	0.052 (11)	−0.059 (12)
O17	0.078 (13)	0.044 (11)	0.075 (13)	−0.002 (10)	−0.033 (10)	−0.003 (9)
O18	0.049 (11)	0.089 (15)	0.044 (10)	0.026 (10)	−0.007 (8)	0.011 (10)
O19A	0.023 (14)	0.030 (15)	0.036 (15)	−0.003 (11)	0.007 (11)	−0.007 (12)
O21A	0.018 (13)	0.035 (15)	0.030 (15)	0.002 (11)	0.007 (11)	0.004 (12)
O19B	0.042 (16)	0.029 (15)	0.019 (14)	0.002 (12)	0.000 (12)	0.002 (11)
O21B	0.029 (15)	0.049 (17)	0.021 (13)	0.001 (12)	0.011 (11)	−0.009 (12)
O1W	0.077 (15)	0.12 (2)	0.084 (16)	0.024 (14)	−0.011 (12)	−0.006 (14)
O2W	0.165 (13)	0.164 (13)	0.191 (13)	−0.005 (3)	0.102 (4)	−0.017 (3)
O3W	0.22 (2)	0.23 (2)	0.39 (2)	0.000 (3)	0.151 (6)	−0.045 (3)
P1	0.026 (4)	0.026 (4)	0.023 (4)	0.001 (3)	0.004 (3)	0.000 (3)

### Geometric parameters (Å, °)

**Table:**

C1—N1	1.37 (3)	Mo2—O15	1.657 (16)
C1—C2	1.34 (3)	Mo2—O16^i^	1.86 (2)
C1—H1	0.9300	Mo2—O11	1.876 (18)
C2—C3	1.35 (4)	Mo2—O14	1.930 (18)
C2—H2	0.9300	Mo2—O12	1.952 (18)
C3—C4	1.36 (4)	Mo2—O19B^i^	2.45 (3)
C3—H3	0.9300	Mo2—O21B	2.48 (3)
C4—C5	1.37 (3)	Mo3—O10	1.655 (16)
C4—H4	0.9300	Mo3—O1	1.831 (17)
C5—N1	1.33 (3)	Mo3—O7	1.903 (16)
C5—C6	1.45 (3)	Mo3—O13^i^	1.866 (17)
C6—N2	1.31 (3)	Mo3—O9	1.969 (18)
C6—C7	1.41 (3)	Mo3—O19A^i^	2.44 (2)
C7—C8	1.42 (4)	Mo3—O21A	2.52 (3)
C7—H7	0.9300	Mo4—O8	1.665 (16)
C8—N3	1.32 (4)	Mo4—O6	1.840 (17)
C8—H8	0.9300	Mo4—O11	1.910 (18)
C9—N4	1.34 (3)	Mo4—O5	1.853 (17)
C9—C10	1.36 (4)	Mo4—O1	1.979 (17)
C9—H9	0.9300	Mo4—O21B	2.46 (3)
C10—C11	1.33 (4)	Mo4—O21A	2.49 (3)
C10—H10	0.9300	Mo5—O18	1.655 (15)
C11—C12	1.36 (4)	Mo5—O4	1.897 (18)
C11—H11	0.9300	Mo5—O5	1.950 (17)
C12—C13	1.35 (4)	Mo5—O12^i^	1.860 (17)
C12—H12	0.9300	Mo5—O7	1.915 (16)
C13—N4	1.34 (3)	Mo5—O19B	2.45 (3)
C13—C14	1.48 (4)	Mo5—O21A	2.52 (2)
C14—N5	1.31 (3)	Mo6—O2	1.658 (16)
C14—C15	1.42 (4)	Mo6—O9^i^	1.828 (18)
C15—C16	1.41 (5)	Mo6—O14	1.854 (17)
C15—H15	0.9300	Mo6—O17	1.914 (17)
C16—N6	1.31 (3)	Mo6—O6	1.990 (17)
C16—H16	0.9300	Mo6—O19A	2.45 (3)
C17—N7	1.35 (3)	Mo6—O21B	2.55 (2)
C17—C18	1.37 (4)	N2—N3	1.37 (3)
C17—H17	0.9300	N3—H3A	0.8600
C18—C19	1.35 (4)	N5—N6	1.36 (3)
C18—H18	0.9300	N6—H6	0.8600
C19—C20	1.41 (4)	N8—N9	1.33 (3)
C19—H19	0.9300	N9—H9A	0.8600
C20—C21	1.36 (4)	O9—Mo6^i^	1.828 (18)
C20—H20	0.9300	O12—Mo5^i^	1.860 (17)
C21—N7	1.33 (3)	O13—Mo3^i^	1.866 (17)
C21—C22	1.47 (3)	O16—Mo2^i^	1.86 (2)
C22—N8	1.36 (3)	O19A—P1	1.57 (3)
C22—C23	1.39 (4)	O19A—O21A^i^	1.75 (4)
C23—C24	1.37 (4)	O19A—O21B	1.81 (4)
C23—H23	0.9300	O19A—O19B	1.86 (4)
C24—N9	1.37 (3)	O19A—Mo3^i^	2.44 (2)
C24—H24	0.9300	O21A—P1	1.49 (2)
Ni1—N5	2.077 (19)	O21A—O21B	1.65 (4)
Ni1—N8	2.06 (2)	O21A—O19B	1.74 (4)
Ni1—N2	2.084 (19)	O21A—O19A^i^	1.75 (4)
Ni1—N4	2.13 (2)	O19B—P1	1.55 (3)
Ni1—N1	2.118 (17)	O19B—O21B^i^	1.76 (3)
Ni1—N7	2.14 (2)	O19B—Mo2^i^	2.45 (3)
Mo1—O3	1.653 (15)	O21B—P1	1.50 (3)
Mo1—O13	1.923 (19)	O21B—O19B^i^	1.76 (3)
Mo1—O16	1.91 (2)	P1—O21A^i^	1.49 (2)
Mo1—O17	1.911 (18)	P1—O21B^i^	1.50 (3)
Mo1—O4	1.919 (19)	P1—O19B^i^	1.55 (3)
Mo1—O19A	2.49 (3)	P1—O19A^i^	1.57 (3)
Mo1—O19B	2.50 (3)		
			
N1—C1—C2	121 (2)	O4—Mo5—O19B	64.3 (9)
N1—C1—H1	119.2	O5—Mo5—O19B	92.8 (9)
C2—C1—H1	119.6	O12^i^—Mo5—O19B	64.0 (9)
C3—C2—C1	120 (2)	O7—Mo5—O19B	93.4 (9)
C3—C2—H2	120.0	O18—Mo5—O21A	159.4 (9)
C1—C2—H2	119.7	O4—Mo5—O21A	90.7 (10)
C2—C3—C4	120 (2)	O5—Mo5—O21A	63.2 (8)
C2—C3—H3	120.3	O12^i^—Mo5—O21A	93.3 (10)
C4—C3—H3	120.1	O7—Mo5—O21A	64.7 (8)
C3—C4—C5	119 (3)	O19B—Mo5—O21A	41.0 (8)
C3—C4—H4	120.6	O2—Mo6—O9^i^	103.8 (10)
C5—C4—H4	120.5	O2—Mo6—O14	103.0 (10)
N1—C5—C4	122 (2)	O9^i^—Mo6—O14	91.9 (9)
N1—C5—C6	115.1 (19)	O2—Mo6—O17	100.1 (10)
C4—C5—C6	123 (2)	O9^i^—Mo6—O17	90.1 (7)
N2—C6—C7	111 (2)	O14—Mo6—O17	155.6 (10)
N2—C6—C5	117 (2)	O2—Mo6—O6	99.5 (9)
C7—C6—C5	132 (2)	O9^i^—Mo6—O6	156.5 (10)
C6—C7—C8	104 (3)	O14—Mo6—O6	85.9 (7)
C6—C7—H7	128.3	O17—Mo6—O6	82.7 (8)
C8—C7—H7	128.1	O2—Mo6—O19A	159.8 (9)
N3—C8—C7	107 (3)	O9^i^—Mo6—O19A	63.8 (9)
N3—C8—H8	126.4	O14—Mo6—O19A	93.7 (10)
C7—C8—H8	126.1	O17—Mo6—O19A	65.6 (9)
N4—C9—C10	122 (3)	O6—Mo6—O19A	92.9 (8)
N4—C9—H9	118.5	O2—Mo6—O21B	157.3 (9)
C10—C9—H9	119.4	O9^i^—Mo6—O21B	95.4 (10)
C11—C10—C9	120 (3)	O14—Mo6—O21B	63.8 (9)
C11—C10—H10	120.2	O17—Mo6—O21B	91.8 (10)
C9—C10—H10	120.2	O6—Mo6—O21B	62.7 (8)
C10—C11—C12	121 (3)	O19A—Mo6—O21B	42.5 (9)
C10—C11—H11	119.00	C5—N1—C1	117.9 (19)
C12—C11—H11	119.0	C5—N1—Ni1	114.9 (14)
C11—C12—C13	117 (3)	C1—N1—Ni1	127.2 (15)
C11—C12—H12	121.4	C6—N2—N3	106.7 (19)
C13—C12—H12	121.5	C6—N2—Ni1	115.7 (15)
N4—C13—C12	124 (3)	N3—N2—Ni1	137.5 (16)
N4—C13—C14	114 (2)	C8—N3—N2	111 (2)
C12—C13—C14	122 (2)	C8—N3—H3A	125.00
N5—C14—C15	111 (3)	N2—N3—H3A	124.9
N5—C14—C13	117 (2)	C13—N4—C9	116 (2)
C15—C14—C13	132 (3)	C13—N4—Ni1	115.1 (18)
C14—C15—C16	103 (3)	C9—N4—Ni1	128.6 (17)
C14—C15—H15	128.7	C14—N5—N6	105 (2)
C16—C15—H15	128.1	C14—N5—Ni1	116.7 (18)
N6—C16—C15	107 (3)	N6—N5—Ni1	137.8 (19)
N6—C16—H16	126.0	N5—N6—C16	113 (3)
C15—C16—H16	127.2	N5—N6—H6	123.1
N7—C17—C18	124 (3)	C16—N6—H6	123.7
N7—C17—H17	118.2	C21—N7—C17	117 (2)
C18—C17—H17	118.0	C21—N7—Ni1	115.7 (16)
C19—C18—C17	119 (3)	C17—N7—Ni1	127.6 (19)
C19—C18—H18	120.8	C22—N8—N9	106 (2)
C17—C18—H18	120.7	C22—N8—Ni1	115.4 (16)
C18—C19—C20	120 (3)	N9—N8—Ni1	138.5 (17)
C18—C19—H19	120.9	C24—N9—N8	111 (2)
C20—C19—H19	121.00	C24—N9—H9A	124.5
C21—C20—C19	117 (3)	N8—N9—H9A	124.7
C21—C20—H20	120.8	Mo3—O1—Mo4	138.2 (11)
C19—C20—H20	121.8	Mo5—O4—Mo1	139.5 (12)
N7—C21—C20	124 (3)	Mo5—O5—Mo4	140.4 (11)
N7—C21—C22	114 (2)	Mo4—O6—Mo6	138.9 (11)
C20—C21—C22	122 (3)	Mo3—O7—Mo5	139.2 (10)
N8—C22—C23	110 (2)	Mo6^i^—O9—Mo3	139.6 (13)
N8—C22—C21	117 (2)	Mo4—O11—Mo2	140.0 (12)
C23—C22—C21	133 (2)	Mo5^i^—O12—Mo2	138.5 (12)
C22—C23—C24	105 (2)	Mo1—O13—Mo3^i^	140.7 (13)
C22—C23—H23	127.8	Mo6—O14—Mo2	141.1 (11)
C24—C23—H23	126.9	Mo2^i^—O16—Mo1	142.1 (13)
N9—C24—C23	108 (2)	Mo1—O17—Mo6	136.8 (11)
N9—C24—H24	127.0	P1—O19A—O21A^i^	53.0 (11)
C23—C24—H24	125.4	P1—O19A—O21B	52.0 (11)
N5—Ni1—N8	95.9 (9)	O21A^i^—O19A—O21B	88.4 (17)
N5—Ni1—N2	167.4 (8)	P1—O19A—O19B	53.1 (11)
N8—Ni1—N2	93.7 (8)	O21A^i^—O19A—O19B	87.3 (16)
N5—Ni1—N4	77.1 (9)	O21B—O19A—O19B	85.4 (16)
N8—Ni1—N4	170.8 (8)	P1—O19A—Mo3^i^	124.6 (14)
N2—Ni1—N4	94.1 (8)	O21A^i^—O19A—Mo3^i^	71.6 (12)
N5—Ni1—N1	94.5 (7)	O21B—O19A—Mo3^i^	136.6 (16)
N8—Ni1—N1	91.8 (7)	O19B—O19A—Mo3^i^	129.8 (15)
N2—Ni1—N1	77.1 (7)	P1—O19A—Mo6	123.7 (14)
N4—Ni1—N1	94.8 (7)	O21A^i^—O19A—Mo6	132.5 (16)
N5—Ni1—N7	95.3 (7)	O21B—O19A—Mo6	71.7 (11)
N8—Ni1—N7	77.6 (8)	O19B—O19A—Mo6	131.4 (14)
N2—Ni1—N7	94.7 (7)	Mo3^i^—O19A—Mo6	93.6 (9)
N4—Ni1—N7	96.9 (8)	P1—O19A—Mo1	121.6 (13)
N1—Ni1—N7	166.2 (7)	O21A^i^—O19A—Mo1	132.3 (16)
O3—Mo1—O13	102.6 (10)	O21B—O19A—Mo1	127.5 (15)
O3—Mo1—O16	102.1 (10)	O19B—O19A—Mo1	68.5 (11)
O13—Mo1—O16	87.4 (9)	Mo3^i^—O19A—Mo1	92.6 (9)
O3—Mo1—O17	102.4 (9)	Mo6—O19A—Mo1	92.0 (9)
O13—Mo1—O17	88.0 (7)	P1—O21A—O21B	56.6 (12)
O16—Mo1—O17	155.5 (10)	P1—O21A—O19B	56.8 (12)
O3—Mo1—O4	101.8 (9)	O21B—O21A—O19B	94.2 (18)
O13—Mo1—O4	155.5 (10)	P1—O21A—O19A^i^	57.1 (12)
O16—Mo1—O4	85.9 (8)	O21B—O21A—O19A^i^	92.9 (17)
O17—Mo1—O4	88.3 (9)	O19B—O21A—O19A^i^	91.6 (17)
O3—Mo1—O19A	159.1 (9)	P1—O21A—Mo3	124.1 (14)
O13—Mo1—O19A	62.4 (9)	O21B—O21A—Mo3	128.9 (16)
O16—Mo1—O19A	92.1 (10)	O19B—O21A—Mo3	130.8 (16)
O17—Mo1—O19A	64.6 (8)	O19A^i^—O21A—Mo3	67.1 (12)
O4—Mo1—O19A	94.3 (9)	P1—O21A—Mo4	126.1 (14)
O3—Mo1—O19B	157.2 (9)	O21B—O21A—Mo4	69.5 (13)
O13—Mo1—O19B	93.2 (10)	O19B—O21A—Mo4	130.8 (16)
O16—Mo1—O19B	62.0 (9)	O19A^i^—O21A—Mo4	133.7 (16)
O17—Mo1—O19B	94.3 (9)	Mo3—O21A—Mo4	90.7 (8)
O4—Mo1—O19B	62.9 (9)	P1—O21A—Mo5	124.0 (14)
O19A—Mo1—O19B	43.6 (8)	O21B—O21A—Mo5	134.5 (16)
O15—Mo2—O16^i^	102.2 (10)	O19B—O21A—Mo5	67.3 (12)
O15—Mo2—O11	100.8 (10)	O19A^i^—O21A—Mo5	127.1 (15)
O16^i^—Mo2—O11	91.7 (9)	Mo3—O21A—Mo5	90.4 (8)
O15—Mo2—O14	102.3 (9)	Mo4—O21A—Mo5	91.1 (8)
O16^i^—Mo2—O14	155.2 (10)	P1—O19B—O21A	53.3 (12)
O11—Mo2—O14	87.5 (8)	P1—O19B—O21B^i^	53.3 (12)
O15—Mo2—O12	103.3 (10)	O21A—O19B—O21B^i^	90.3 (17)
O16^i^—Mo2—O12	86.7 (8)	P1—O19B—O19A	53.8 (12)
O11—Mo2—O12	155.6 (10)	O21A—O19B—O19A	88.1 (16)
O14—Mo2—O12	83.9 (8)	O21B^i^—O19B—O19A	86.0 (16)
O15—Mo2—O19B^i^	159.6 (9)	P1—O19B—Mo2^i^	123.3 (13)
O16^i^—Mo2—O19B^i^	63.8 (10)	O21A—O19B—Mo2^i^	135.8 (17)
O11—Mo2—O19B^i^	94.5 (10)	O21B^i^—O19B—Mo2^i^	70.1 (12)
O14—Mo2—O19B^i^	91.5 (9)	O19A—O19B—Mo2^i^	127.6 (15)
O12—Mo2—O19B^i^	63.0 (9)	P1—O19B—Mo5	125.1 (14)
O15—Mo2—O21B	158.5 (9)	O21A—O19B—Mo5	71.8 (12)
O16^i^—Mo2—O21B	93.1 (10)	O21B^i^—O19B—Mo5	133.9 (17)
O11—Mo2—O21B	63.2 (9)	O19A—O19B—Mo5	133.4 (15)
O14—Mo2—O21B	64.5 (8)	Mo2^i^—O19B—Mo5	93.4 (9)
O12—Mo2—O21B	92.5 (10)	P1—O19B—Mo1	121.6 (15)
O19B^i^—Mo2—O21B	41.9 (8)	O21A—O19B—Mo1	128.9 (15)
O10—Mo3—O1	103.0 (9)	O21B^i^—O19B—Mo1	129.5 (16)
O10—Mo3—O7	101.0 (9)	O19A—O19B—Mo1	67.9 (12)
O1—Mo3—O7	89.5 (7)	Mo2^i^—O19B—Mo1	92.1 (9)
O10—Mo3—O13^i^	101.9 (11)	Mo5—O19B—Mo1	92.5 (8)
O1—Mo3—O13^i^	91.7 (8)	P1—O21B—O21A	56.1 (13)
O7—Mo3—O13^i^	156.1 (10)	P1—O21B—O19B^i^	56.2 (12)
O10—Mo3—O9	100.3 (10)	O21A—O21B—O19B^i^	93.6 (17)
O1—Mo3—O9	156.6 (10)	P1—O21B—O19A	55.5 (12)
O7—Mo3—O9	84.2 (7)	O21A—O21B—O19A	92.3 (18)
O13^i^—Mo3—O9	85.3 (7)	O19B^i^—O21B—O19A	88.9 (17)
O10—Mo3—O19A^i^	157.4 (9)	P1—O21B—Mo4	127.4 (15)
O1—Mo3—O19A^i^	95.4 (9)	O21A—O21B—Mo4	71.4 (13)
O7—Mo3—O19A^i^	92.0 (9)	O19B^i^—O21B—Mo4	136.7 (17)
O13^i^—Mo3—O19A^i^	64.2 (10)	O19A—O21B—Mo4	130.8 (15)
O9—Mo3—O19A^i^	62.4 (9)	P1—O21B—Mo2	124.2 (14)
O10—Mo3—O21A	160.9 (9)	O21A—O21B—Mo2	132.5 (17)
O1—Mo3—O21A	65.6 (8)	O19B^i^—O21B—Mo2	68.0 (12)
O7—Mo3—O21A	65.0 (8)	O19A—O21B—Mo2	128.7 (15)
O13^i^—Mo3—O21A	93.9 (10)	Mo4—O21B—Mo2	92.1 (9)
O9—Mo3—O21A	91.4 (10)	P1—O21B—Mo6	121.2 (15)
O19A^i^—Mo3—O21A	41.3 (8)	O21A—O21B—Mo6	132.8 (16)
O8—Mo4—O6	103.5 (10)	O19B^i^—O21B—Mo6	125.1 (16)
O8—Mo4—O11	102.2 (10)	O19A—O21B—Mo6	65.8 (11)
O6—Mo4—O11	88.7 (7)	Mo4—O21B—Mo6	91.5 (8)
O8—Mo4—O5	101.7 (10)	Mo2—O21B—Mo6	90.3 (9)
O6—Mo4—O5	92.4 (8)	O21A—P1—O21A^i^	180 (3)
O11—Mo4—O5	155.2 (10)	O21A—P1—O21B	67.3 (14)
O8—Mo4—O1	99.8 (9)	O21A^i^—P1—O21B	112.7 (14)
O6—Mo4—O1	156.4 (9)	O21A—P1—O21B^i^	112.7 (14)
O11—Mo4—O1	82.6 (8)	O21A^i^—P1—O21B^i^	67.3 (14)
O5—Mo4—O1	86.6 (7)	O21B—P1—O21B^i^	180.0 (19)
O8—Mo4—O21B	161.4 (9)	O21A—P1—O19B	69.9 (14)
O6—Mo4—O21B	66.3 (8)	O21A^i^—P1—O19B	110.1 (14)
O11—Mo4—O21B	63.4 (9)	O21B—P1—O19B	109.4 (14)
O5—Mo4—O21B	94.4 (9)	O21B^i^—P1—O19B	70.6 (14)
O1—Mo4—O21B	90.2 (8)	O21A—P1—O19B^i^	110.1 (14)
O8—Mo4—O21A	159.0 (9)	O21A^i^—P1—O19B^i^	69.9 (14)
O6—Mo4—O21A	93.7 (9)	O21B—P1—O19B^i^	70.6 (14)
O11—Mo4—O21A	90.1 (9)	O21B^i^—P1—O19B^i^	109.4 (14)
O5—Mo4—O21A	65.1 (8)	O19B—P1—O19B^i^	180 (2)
O1—Mo4—O21A	64.6 (8)	O21A—P1—O19A	110.1 (13)
O21B—Mo4—O21A	39.1 (8)	O21A^i^—P1—O19A	69.9 (13)
O18—Mo5—O4	102.1 (10)	O21B—P1—O19A	72.5 (14)
O18—Mo5—O5	101.5 (9)	O21B^i^—P1—O19A	107.5 (14)
O4—Mo5—O5	85.3 (8)	O19B—P1—O19A	73.1 (14)
O18—Mo5—O12^i^	102.6 (10)	O19B^i^—P1—O19A	106.9 (14)
O4—Mo5—O12^i^	89.9 (8)	O21A—P1—O19A^i^	69.9 (13)
O5—Mo5—O12^i^	155.9 (10)	O21A^i^—P1—O19A^i^	110.1 (13)
O18—Mo5—O7	102.1 (9)	O21B—P1—O19A^i^	107.5 (14)
O4—Mo5—O7	155.3 (9)	O21B^i^—P1—O19A^i^	72.5 (14)
O5—Mo5—O7	85.4 (7)	O19B—P1—O19A^i^	106.9 (14)
O12^i^—Mo5—O7	89.3 (8)	O19B^i^—P1—O19A^i^	73.1 (14)
O18—Mo5—O19B	159.6 (10)	O19A—P1—O19A^i^	180 (2)

Symmetry codes: (i) −*x*+1/2, −*y*+1/2, −*z*.

### Hydrogen-bond geometry (Å, °)

**Table:**

*D*—H···*A*	*D*—H	H···*A*	*D*···*A*	*D*—H···*A*
N3—H3A···O17^ii^	0.86	2.05	2.83 (3)	149
N6—H6···O2W	0.86	1.99	2.84 (5)	166
N9—H9A···O1W	0.86	1.92	2.74 (3)	160

Symmetry codes: (ii) −*x*+1/2, *y*+1/2, −*z*+1/2.

## References

[bb1] Artero, V. & Proust, A. (2000). *Eur. J. Inorg. Chem.*, pp. 2393–2400

[bb2] Bruker (2001). *SAINT-Plus* and *SADABS* Bruker AXS Inc., Madison, Wisconsin, USA.

[bb3] Bruker (2004). *APEX2* Bruker AXS Inc., Madison, Wisconsin, USA.

[bb4] Hao, L., Liu, T., Chen, J. & Zhang, X. (2010). *Acta Cryst.* E**66**, m283–m284. 10.1107/S1600536810004861 PMC2983704 21580229

[bb5] Hao, L., Ma, C., Chen, J., Zhang, X. & Zhang, X. (2010). *Acta Cryst.* E**66**, m231–m232. 10.1107/S160053681000320X PMC2979847 21579687

[bb6] Hao, L., Wang, Y., Zhang, X., Chen, J. & Zhang, X. (2010). *Acta Cryst.* E**66**, m268–m269. 10.1107/S1600536810004307 PMC2983543 21580219

[bb7] Kurmoo, M., Bonamico, M., Bellitto, C., Fares, V., Federici, F., Guionneau, P., Ducasse, L., Kitagawa, H. & Day, P. (1998). *Adv. Mater.* **7**, 545–550.

[bb8] Niu, J. Y., Shan, B. Z. & You, X. Z. (1999). *Transition Met. Chem.* **24**, 108–114

[bb9] Pope, M. T. & Müller, A. (1991). *Angew. Chem. Int. Ed.* **30**, 34–38.

[bb10] Sheldrick, G. M. (2008). *Acta Cryst.* A**64**, 112–122. 10.1107/S0108767307043930 18156677

[bb11] Yang, W. B., Lu, C. Z., Wu, C. D. & Zhuang, H. H. (2003). *Chin. J. Struct. Chem.* **22**, 137–142.

[bb12] Zhang, X. T., Dou, J. M., Wei, P. H., Li, D. C., Li, B., Shi, C. W. & Hu, B. (2009). *Inorg. Chim. Acta*, **362**, 3325–3332.

[bb13] Zhang, X. T., Wei, P. H., Sun, D. F., Ni, Z. H., Dou, J. M., Li, B., Shi, C. W. & Hu, B. (2009). *Cryst. Growth Des.* **9**, 4424–4428.

